# Complete hematologic response of early T-cell progenitor acute lymphoblastic leukemia to the γ-secretase inhibitor BMS-906024: genetic and epigenetic findings in an outlier case

**DOI:** 10.1101/mcs.a000539

**Published:** 2015-10

**Authors:** Birgit Knoechel, Ami Bhatt, Li Pan, Chandra S. Pedamallu, Eric Severson, Alejandro Gutierrez, David M. Dorfman, Frank C. Kuo, Michael Kluk, Andrew L. Kung, Patrick Zweidler-McKay, Matthew Meyerson, Stephen C. Blacklow, Daniel J. DeAngelo, Jon C. Aster

**Affiliations:** 1 Department of Medical Oncology, Dana-Farber Cancer Institute, Boston, Massachusetts 02215, USA;; 2 Departments of Medicine and Genetics, Stanford University, Stanford, California 95305, USA;; 3 Department of Pathology, Brigham and Women's Hospital, Boston, Massachusetts 02115, USA;; 4 Broad Institute of MIT and Harvard University, Cambridge, Massachusetts 02142, USA;; 5 Division of Hematology/Oncology, Boston Children's Hospital and Dana-Farber Cancer Institute, Boston, Massachusetts 02115, USA;; 6 Department of Pediatrics, Columbia University Medical Center, New York, New York 10032, USA;; 7 Department of Pediatrics, University of Texas MD Anderson Cancer Center, Houston, Texas 77030, USA;; 8 Department of Biochemistry and Molecular Pharmacology, Harvard Medical School, Boston, Massachusetts 02115, USA

**Keywords:** leukemia

## Abstract

Notch pathway antagonists such as γ-secretase inhibitors (GSIs) are being tested in diverse cancers, but exceptional responses have yet to be reported. We describe the case of a patient with relapsed/refractory early T-cell progenitor acute lymphoblastic leukemia (ETP-ALL) who achieved a complete hematologic response following treatment with the GSI BMS-906024. Whole-exome sequencing of leukemic blasts revealed heterozygous gain-of-function driver mutations in *NOTCH1*, *CSF3R*, and *PTPN11*, and a homozygous/hemizygous loss-of-function mutation in *DNMT3A*. The three gain-of-function mutations were absent from remission marrow cells, but the *DNMT3A* mutation persisted in heterozygous form in remission marrow, consistent with an origin for the patient's ETP-ALL from clonal hematopoiesis. Ex vivo culture of ETP-ALL blasts confirmed high levels of activated NOTCH1 that were repressed by GSI treatment, and RNA-seq documented that GSIs downregulated multiple known Notch target genes. Surprisingly, one potential target gene that was unaffected by GSIs was *MYC*, a key Notch target in GSI-sensitive T-ALL of cortical T-cell type. H3K27ac super-enhancer landscapes near *MYC* showed a pattern previously reported in acute myeloid leukemia (AML) that is sensitive to BRD4 inhibitors, and in line with this ETP-ALL blasts downregulated *MYC* in response to the BRD4 inhibitor JQ1. To our knowledge, this is the first example of complete response of a Notch-mutated ETP-ALL to a Notch antagonist and is also the first description of chromatin landscapes associated with ETP-ALL. Our experience suggests that additional attempts to target Notch in Notch-mutated ETP-ALL are merited.

## INTRODUCTION

The Notch signaling pathway plays a key role in early stages of normal T-cell development and when hyperactivated is a potent inducer of T-cell acute lymphoblastic leukemia (T-ALL) (for recent review, see Van Vlierberghe and Ferrando 2012). The most common mechanism for oncogenic activation of Notch signaling in T-ALL is gain-of-function mutations in *NOTCH1*, which occur in >50% of human T-ALLs. T-ALLs can be subclassified into early T-cell progenitor ALL (ETP-ALL), cortical T-ALL, and mature T-ALL based on stage-specific differentiation markers, with ETP-ALLs being defined by the absence of CD4, CD8, and CD1a and frequent expression of one or more myeloid markers (Coustan-Smith et al. 2009). Early work suggested that NOTCH1 mutations were absent from ETP-ALL; indeed, T-ALLs expressing myeloid markers, a characteristic of ETP-ALL, have been excluded from some trials of Notch pathway inhibitors. However, earlier sequencing studies focused on pediatric ETP-ALL (Zhang et al. 2012), and more recent studies of adult ETP-ALL have shown that NOTCH1 mutations occur in a significant minority of cases (Neumann et al. 2013).

Notch receptors are large multimodular single-pass transmembrane proteins that are activated when they engage ligands of the JAGGED or DELTA families expressed on neighboring cells (for review, see Kopan and Ilagan 2009). Ligand binding alters the conformation of a juxtamembrane negative regulatory region (NRR), rendering Notch sensitive to successive cleavages by ADAM metalloproteases and the intramembranous γ-secretase complex. γ-Secretase cleavage in turn allows the intracellular portion of Notch (ICN) to translocate to the nucleus and form a transcription activation complex with the DNA-binding factor RBPJ and coactivators of the MAML family. The most common NOTCH1 mutations in T-ALL derange the structure of the NRR (Malecki et al. 2006), leading to ligand-independent ICN generation and overexpression of key target genes such as *MYC*, mainly via interaction of ICN/RBPJ complexes with long-range enhancers (Herranz et al. 2014; Yashiro-Ohtani et al. 2014).

The frequent presence of NOTCH1 gain-of-function mutations in T-ALL has provided the rationale for clinical trials of GSIs. Initial trials were plagued by on-target gut toxicity, and the perception that GSI have unacceptable toxicities is still pervasive. However, with intermittent dosing, GSIs can be given safely and have tolerable toxicities, as shown by phase I trials of GSIs in solid tumors (for review, see Aster and Blacklow 2012). What mainly has been lacking is evidence of strong sustained anti-tumor responses. Here we describe the case of a 53-yr-old male with relapsed refractory ETP-ALL who experienced a complete hematologic remission associated with a deep molecular response following treatment with the GSI BMS-906024.

## RESULTS

A male patient presented at the Dana-Farber Cancer Institute (DFCI) at the age of 53 yr with relapsed refractory ETP-ALL. ETP-ALL was first diagnosed 8 mo prior to presentation. At this time, flow cytometric analysis showed a CD45(dim) blast population that was positive for CD34 (84%), CD3 (cytoplasmic, 90%), CD5 (75%), CD7 (86%), CD33 (87%), and TdT(dim, 58%), and negative for CD1a, CD4, and CD8. Cytogenetics revealed a 46,XY karyotype with del(7p13). The patient was treated with induction chemotherapy at an outside institution per the CALGB 9111 regimen (Larson et al. 1998). A repeat marrow examination 4 wk after induction was interpreted as being consistent with remission, and repeat cytogenetic analysis showed a normal 46,XY karyotype. After two cycles of consolidation chemotherapy, the patient was started on methotrexate/mercaptopurine maintenance therapy 6.5 mo after initial presentation. This was complicated by severe neutropenia, necessitating a 50% reduction in the second cycle of maintenance chemotherapy.

A routine follow-up marrow examination before the third cycle of maintenance therapy showed evidence of early relapse of ETP-ALL, and he was referred to the DFCI for further treatment. Two cycles of salvage nelarabine therapy were given, but repeat marrow examination after the second cycle revealed persistent ETP-ALL. Flow cytometric analysis at this time showed CD45(dim) blasts that were positive for CD34 (99%), HLA-DR (10%), CD5 (97%), CD7 (98%), CD13 (98%), CD33 (100%), and TdT (dim, small subset), and negative for CD1a, CD4, CD8, and surface CD3. Cytogenetic analysis again showed a 46,XY karyotype with del(7p13). TCRγ (encoded by *TRG*) gene analysis revealed monoallelic rearrangement of TCRγ (data not shown), a feature also consistent with ETP-ALL that has been associated with high-risk disease (Gutierrez et al. 2010).

**Figure 1.**
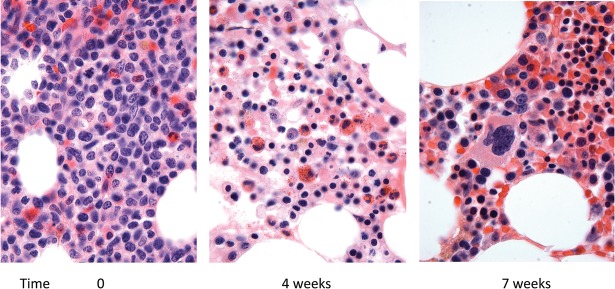
Bone marrow biopsy histology prior to and 4 and 7 wk after initiation of treatment with BMS-906024 (hematoxylin and eosin staining, 400× magnification). The marrow contains mainly blasts prior to treatment that are cleared following treatment.

The patient was enrolled on a trial of BMS-906024, an intravenously administered GSI, at a dose of 6 mg once per week in 4-wk cycles. The patient also received dexamethasone, 20 mg orally, for 4 d with the first dose of BMS-906024 during the first and second cycles of BMS-906024 treatment. The first 4-wk cycle of BMS-906024 was complicated by severe thrombocytopenia, which necessitated holding BMS-906024 for 1 wk after the second dose, after which treatment resumed. By the end the first 4-wk cycle of therapy, blasts were cleared from the peripheral blood (Table 1) and bone marrow (Fig. 1). By the end of the second 4-wk cycle of BMS-906024, peripheral blood counts had normalized (Table 1), the marrow was normocellular and showed maturing trilineage hematopoiesis (Fig. 1), and minimal residual disease testing by flow cytometry was negative (1 × 10^6^ events evaluated). Cytogenetic analysis revealed a normal 46,XY karyotype.

**Table 1.** Hematologic response following initiation of BMS-906024 treatment

Days	0	7	14	21	28	35	42	50	56	63	76	83	101
GSI	+	+		+	+	+	+	+	+	+	+		
DEX	+				+								
WBC	46	14.7	9.3	0.9	0.9	1.6	1.6	2.2	3.9	4.1	6.3	8.9	6.4
Blasts	85%	80%	46%	–	–	–	–	–	–	–	–	–	–
Hgb	9.9	10.8	10.1	7.4	8.5	10.5	8.9	10.8	11.3	11.8	12	12.9	12
Plt	42	13	7	4	9	22	50	91	117	109	179	166	163
MRD					Neg*				Neg**				

GSI, days on which BMS-906024 was administered; DEX, starting point of 4 d cycles of dexamethasone; WBC, white blood cell count, 10^3^/mm^3^; blasts, peripheral blood blasts; Hgb, hemoglobin, gm/dL; Plt, platelet count, 10^3^/mm^3^; MRD, minimal residual disease by flow cytometry; Neg*, no leukemic blasts detected in 1.2 × 10^5^ events; Neg**, no leukemic blasts detected, 1 × 10^6^ events.

The patient subsequently received one additional 4-wk cycle of BMS-906024 without complication, and then was removed from protocol in preparation for hematopoietic stem cell transplantation from a matched unrelated donor. Conditioning for transplantation consisted of Cytoxan, 1800 mg/m^2^, for 2 d and 1200 cGy of total body irradiation in six fractionated doses. The posttransplant course was complicated by grade 4 skin and ocular acute graft-versus-host disease (GVHD) and subsequently by moderately severe skin and ocular chronic GVHD. He is now 19 mo posttransplant with no evidence of leukemia.

To gain insight into the basis of this tumor's response to BMS-906024, we initially performed targeted next-generation sequencing (NGS) using a previously reported gene panel (Wagle et al. 2012) and subsequently extended this to whole-exome NGS (WES). Both sequencing analyses revealed driver mutations in *NOTCH1*, *PTPN11*, and *DNMT3A*, and WES revealed an additional driver mutation in *CSF3R* (Table 2), a gene that was not covered by the targeted gene panel used. All four driver mutations were subsequently confirmed by direct Sanger sequencing of PCR products (Supplemental Fig. 1). Based on the variant allele fraction, the *CSF3R* mutation was present at heterozygous dosage and resulted in a T618I substitution. This mutation has been described in a high fraction of chronic neutrophilic leukemia and produces constitutive activation of CSF3R (also known as the G-CSF receptor) (Maxson et al. 2013). The *PTPN11* mutation also appeared to be heterozygous based on variant allele fraction and resulted in a F285S substitution that corresponds to a gain-of-function mutation implicated in a subset of Noonan syndrome (Tartaglia and Gelb 2005). Virtually all *DNMT3A* sequence reads were mutant; based on lack of evidence of copy-number variation in the region of Chromosome 1 containing *DNMT3A* (described below), this mutation appears to be homozygous and creates a nonsense mutation at codon 402. The encoded mutated polypeptide, DNMT3A Q402*, lacks the carboxy-terminal plant homeodomain and catalytic domain that are required for DNMT3A function. Consistent with the diagnosis of ETP-ALL, gain-of-function *CSF3R* and *PTPN11* mutations and loss of function *DNMT3A* mutations have been described in ETP-ALL (Coustan-Smith et al. 2009; Zhang et al. 2012; Maxson et al. 2013), but not, to the best of our knowledge, in cortical or mature T-ALL.

**Table 2.** Genomic alterations identified by next-generation sequencing

Gene	Chrom.	HGVS DNA Ref.	HGVS protein ref.	Variant allele fraction (%)	Fold coverage	Effect
Targeted-exon sequencing (leukemia, bone marrow aspirate, 85% blasts)						
*NOTCH1*	9	c.4775T>G	p.F1592C	64.9	165	GOF
*PTPN11*	12	c.854T>C	p.F285S	40.0	115	GOF
*DNMT3A*	2	c.1204C>T	p.Q402*	99.3	139	LOF
Whole-exome sequencing (leukemia, bone marrow aspirate, 85% blasts)						
*NOTCH1*	9	c.4775T>G	p.F1592C	70.5	94	GOF
*PTPN11*	12	c.854T>C	p.F285S	45.7	142	GOF
*DNMT3A*	2	c.1204C>T	p.Q402*	98.7	89	LOF
*CSF3R*	1	c.1853C>T	pT618I	46.2	97	GOF
Whole-exome sequencing (remission marrow, no identified lymphoblasts)						
*NOTCH1*	9	WT			48	
*PTPN11*	12	WT			108	
*DNMT3A*	2	c.1204C>T	p.Q402*	54.6	67	LOF
*CSF3R*	1	WT			76	

HGVS, Human Genome Variation Society; GOF, gain of function; LOF, loss of function; WT, wild type.

**Figure 2.**
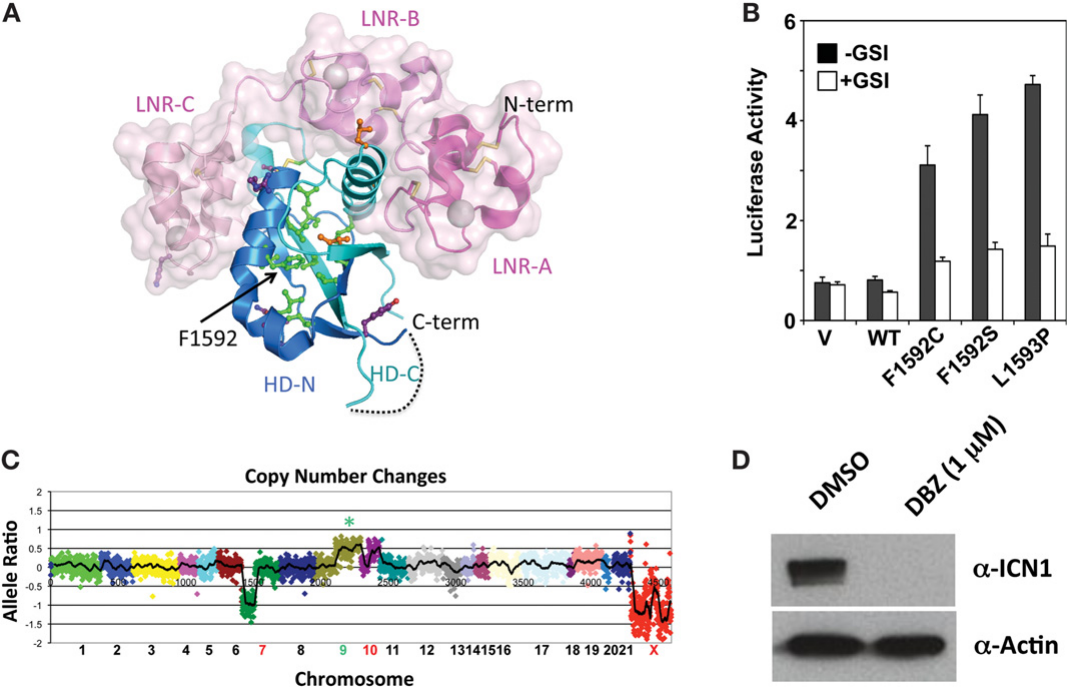
Characterization of the NOTCH1 gain-of-function mutation F1592C. (*A*) Position of F1592C in the NOTCH1 negative regulatory region (NRR). Mapping is based on the crystallographic structure of Gordon et al. (2009b). Residues previously reported be mutated in T-ALL are shown in green (core residues), orange (interdomain interface residues), or purple (polar residues). The three LNR repeats are colored various shades of pink with a translucent surface, and the core “heterodimerization domain” is colored blue prior to the site of furin cleavage (HD-N) and cyan thereafter (HD-C). F1592 sits in the center of the hydrophobic core of the heterodimerization domain. The image is rendered with the program Pymol and is adapted from Gordon et al. (2009a). (*B*) F1592C leads to ligand-independent activation of NOTCH1. The ability of the F1592C variant to activate a Notch firefly luciferase reporter gene was compared with wild-type NOTCH1 and two previously characterized gain-of-function mutants, F1592S and L1593P. Firefly luciferase activity was normalized to an internal *Renilla* luciferase control reporter gene. Normalized firefly luciferase activity is expressed related to the activity of wild-type NOTCH1, which is arbitrarily set to a value of 1. Each expression plasmid was tested in three independent experiments; error bars represent the standard deviations. (*C*) Copy-number gains and losses. The copy number of chromosomal regions was determined from sequencing data as described (Wagle et al. 2012). (*D*) Effect of the GSI diaminobenzidine (DBZ) on activated NOTCH1 (ICN1) levels in leukemic blasts. Blasts were treated with vehicle (DMSO [dimethyl sulfoxide]) or DBZ for 3 d prior to harvest and preparation of whole-cell lysates. A representative Western blot stained for activated NOTCH1 (ICN1) and β-actin is shown.

The *NOTCH1* mutation is predicted to create a F1592C substitution in the NOTCH1 negative regulatory region (NRR) (Fig. 2A), which is the most common site of NOTCH1 gain-of-function mutations in T-ALL and ETP-ALL. Because the F1592C mutation has not been described, we scored this mutant in functional studies using a standard Notch reporter gene assay in which NRR mutants are expressed in a form of NOTCH1 lacking the ligand-binding region of the receptor (Malecki et al. 2006), enabling measurement of the effects of various sequence variants on ligand-independent NOTCH1 activation. This assay confirmed that F1592C causes ligand-independent γ-secretase-dependent activation of NOTCH1 signaling (Fig. 2B). We also used the NGS data to determine genomic copy-number changes. This revealed the 7p deletion noted by karyotyping and a previously unrecognized 10p deletion and a single copy gain involving 9q, including the region encompassing the *NOTCH1* locus (Fig. 2C), an event that has been reported in T-ALL (van Vlierberghe et al. 2006). In this case, the 9q duplication involved the mutated *NOTCH1* allele, as the variant allele:WT allele read ratio was ∼2:1 in both sequencing analyses (Table 2). In line with these observations, studies performed in vitro prior to initiation of BMS-906024 therapy showed that the leukemic blasts contained high levels of activated NOTCH1 (ICN1) that were markedly decreased by treatment with the GSI DBZ (Fig. 2D).

**Figure 3.**
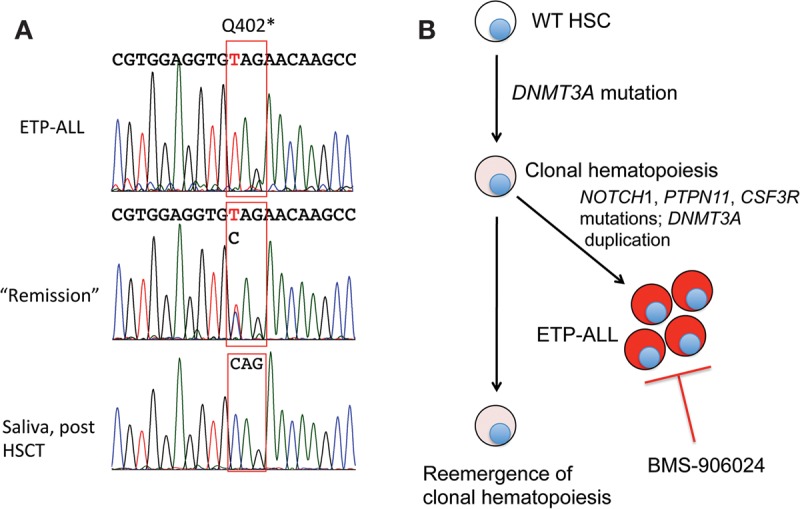
Evidence of ETP-ALL origin from clonal hematopoiesis. (*A*) Sanger sequencing traces of *DNMT3A*. Traces were obtained from DNA obtained from blasts prior to treatment, remission marrow, and saliva following hematopoietic stem cell transplantation. (*B*) Reconstructed clonal evolution of ETP-ALL, based on sequencing of *DNMT3A* as shown in *A*.

To further investigate the nature of this patient's tumor and its response to BMS-906024, we also performed WES on remission marrow obtained after cycle 2 of GSI. This showed wild-type reads for *NOTCH1*, *PTPN11*, and *CSF3R*, and persistence of the *DNMT3A* mutation at heterozygous dosage. To exclude the possibility of a germline heterozygous *DNMT3A* mutation we performed Sanger sequencing on buccal mucosal DNA obtained from the patient following allogeneic transplantation (Fig. 3A). This revealed only wild-type *DNMT3A* reads, in contrast to analyses done on DNA prepared from ETP-ALL blasts and remission marrow pretransplant, which confirmed the presence of homozygous and heterozygous DNMT3A codon 402 mutations, respectively (Fig. 3A). These findings are consistent with a scenario in which ETP-ALL arose out of a background of clonal hematopoiesis associated with a heterozygous *DNMT3A* mutation, a relationship revealed by clearance of ETP-ALL following treatment with BMS-906024 (Fig. 3B).

**Figure 4.**
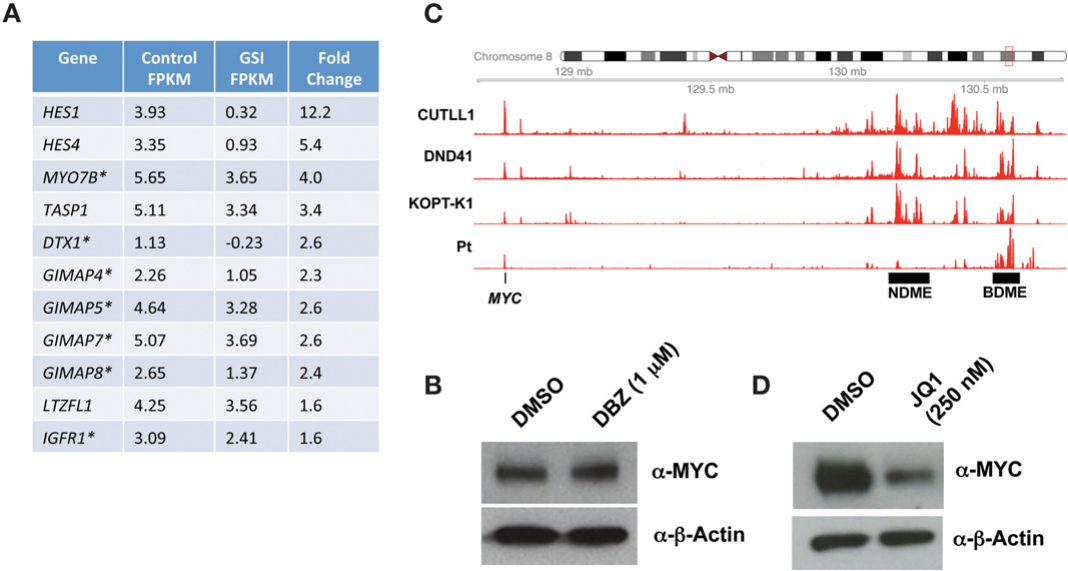
Characterization of likely Notch target genes and *MYC* in ETP-ALL. (*A*) Genes downregulated by the GSI DBZ (1 μm) in ETP-ALL blasts. Cells were treated with DBZ or control (DMSO) for 3 d followed by harvest and analysis by RNA sequencing. FPKM represents the log_2_ value of the FPKM (fragments per transcript kilobase per million fragments mapped). (*B*) Effect of the GSI DBZ on MYC levels in leukemic blasts. Blasts were treated with vehicle (DMSO) or DBZ for 3 d prior to harvest and preparation of whole-cell lysates. A representative Western blot stained for MYC and β-actin is shown. (*C*) H3K27ac ChIP-seq landscapes near *MYC*. Aligned reads are shown for leukemic blasts (Pt) and three NOTCH1-mutated T-ALLs, DND41, KOPT-K1, and CUTLL1, that downregulate MYC in response to GSI treatment. (NDME) Notch-dependent *MYC* enhancer; (BDME) BRD4-dependent *MYC* enhancer. (*D*) Effect of the bromodomain inhibitor JQ1 on MYC levels in leukemic blasts. Blasts were treated with vehicle (DMSO) or JQ1 for 3 d prior to harvest and preparation of whole-cell lysates. A representative Western blot stained for MYC and β-actin is shown.

To identify likely NOTCH1 target genes, we next performed RNA-seq on blasts treated with GSI or vehicle (DMSO) (Fig. 4A). The genes that were most sensitive to GSI included multiple Notch target genes previously identified in cortical T-ALL cells, many of which are regulated by long-range enhancers (Wang et al. 2014). Surprisingly, *MYC*, an important Notch target gene in cortical T-ALL, was not downregulated by GSI treatment at the level of RNA or protein, as Western blotting showed no change in MYC protein despite a sharp decreased in ICN1 levels following GSI treatment (Fig. 4B). We and others recently described a complex multidomain enhancer region 3′ of *MYC* (Herranz et al. 2014; Yashiro-Ohtani et al. 2014). In cortical T-ALL cells in which *MYC* is a direct target of Notch, *MYC* transcription is regulated by a conserved CSL/NOTCH1 binding site within the 3′ enhancer region that lies ∼1.3 Mb from the *MYC* promoter. This enhancer element, termed the Notch-dependent Myc enhancer (NDME), is required for thymocyte development and for induction of T-ALL by Notch in mice (Herranz et al. 2014). To understand the lack of responsiveness of *MYC* to Notch inhibition in this case, we performed H3K27ac chromatin immunoprecipitation sequencing (ChIP-seq) on blasts obtained prior to BMS-906024 treatment (Fig. 4C). This revealed that the NDME was inactive, being largely lacking in H3K27ac marks. In contrast, high levels of H3K27ac marks were observed in a more distal 3′ region termed the BRD4-dependent *MYC* enhancer (BDME), which has been implicated in regulation of *MYC* in acute myeloid leukemia (AML) (Shi et al. 2013) and in GSI-resistant cortical T-ALL (Yashiro-Ohtani et al. 2014). In line with these observations, MYC protein levels in patient blasts were downregulated by the BRD4 inhibitor JQ1 (Fig. 4D). Thus, the *MYC* enhancer state in this GSI-responsive ETP-ALL resembles that seen in AML rather than typical cortical-type T-ALL.

**Figure 5.**
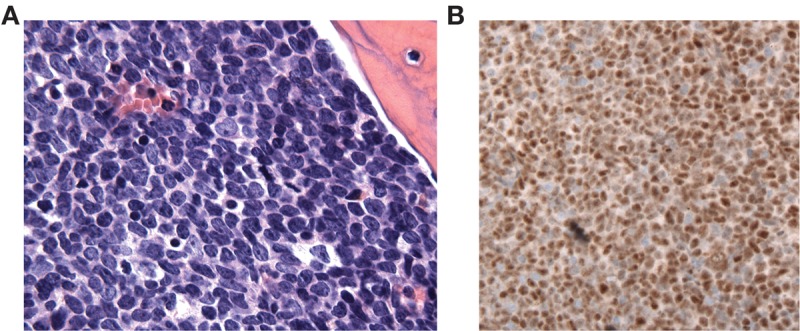
Characterization of ETP-ALL grafts in NSG-S mice (NSG mice expressing IL-3, GM-CSF, and KIT ligand). (*A*) Bone marrow histology (hematoxylin and eosin staining, 400× magnification). (*B*) Immunohistochemical staining for activated NOTCH1 (ICN1). Brown nuclear color denotes specific staining; hematoxylin was used as a counterstain.

Additional work done to try to further evaluate the basis for the response to GSI in this case included attempts to engraft NSG (NOD *scid* γ) and NSG-S mice (NSG mice expressing IL-3, GM-CSF, and KIT ligand) with patient blasts administered by tail vein injection. No growth was observed in NSG mice. In contrast, leukemic blasts grew out in three of three NSG-S primary recipient mice 6 mo after injection (Fig. 5A). Immunohistochemistry revealed that the blasts had detectable ICN1 (Fig. 5B), Sanger sequencing of DNA prepared from leukemic blasts confirmed that the tumor maintained the same genotype (data not shown), and flow cytometry confirmed an immunophenotype consistent with ETP-ALL, as the blasts were CD45(dim), CD34+, HLA-DR+, CD3+, CD5+, CD7+, CD13+, and CD33+. Unfortunately, the leukemic blasts failed to grow when reinjected into secondary recipient animals, precluding further analysis.

## DISCUSSION

Increasing attention is being paid to so-called “super-responders,” outlier cases identified within the context of clinical trials in which tumors demonstrate profound responses to targeted therapies (Mehra et al. 2015). We report one such case, a relapsed refractory ETP-ALL arising out of a background of clonal hematopoiesis that demonstrated a complete response to the GSI BMS-906024. To our knowledge, this is the first patient with ETP-ALL who has been treated with a GSI. ETP-ALL is generally been considered to have a poorer prognosis than other T-ALL subtypes, and relapsed refractory disease is rarely curable, even with stem cell transplantation, highlighting the exceptional nature of this case.

Because we were unable to establish a stable patient-derived xenograft from this tumor, we can only speculate on the basis for the response of this tumor to BMS-906024. Although γ-secretase cleaves multiple surface receptors, the presence of a GSI-targetable NOTCH1 gain-of-function mutant associated with high levels of activated NOTCH1 in the leukemic blasts makes it likely that inhibition of Notch signaling was at least partially culpable for the response to BMS-906024 treatment. It has been reported that Notch signaling renders T-ALL cells resistant to killing by glucocorticoids and conversely that GSIs sensitize T-ALL cells to glucocorticoids (Deftos et al. 1998; Real et al. 2009). It is worth considering if this interaction might have contributed to the extraordinary responsiveness of this case of ETP-ALL, given that the first doses of BMS-906024 in cycles 1 and 2 were given along with 4 d of dexamethasone. We cannot exclude this possibility, but in short-term culture assays we did not observe any increased killing of patient blasts by dexamethasone in the presence of GSI (data not shown). We also note that following initiation of BMS-906024 blasts disappeared from the peripheral blood gradually over a period of 2–3 wk, without evidence of tumor lysis. Alternatively, *HES1*, the most sensitive gene to Notch inhibition in this patient's blasts, has been implicated in maintenance of stemness in a number of cancers (Liu et al. 2015), suggesting that its downregulation, alone or in combination with other targets such as *HES4*, might have led to clearance of leukemic blasts through differentiation. Study of additional responsive cases and establishment of mouse models will be needed to distinguish among these possibilities.

Unexpectedly, one Notch target gene that is strongly correlated with response to Notch inhibition in cortical T-ALL, *MYC*, was not under the control of Notch in this tumor. Lack of response of *MYC* to Notch inhibition can be explained by the inactivate state of the Notch-dependent *MYC* long-range enhancer (NDME) in this tumor, which appears instead to rely on a BRD4-dependent long-range enhancer (BDME) defined previously in AML (Shi et al. 2013). It will be of interest to determine if ETP-ALL generally relies on the BDME to drive *MYC*, as this would provide a rationale for treatment of ETP-ALL will BRD4 inhibitors, which are now being tested in clinical trials.

The other oncogenic driver mutations in this case also merit comment. The *CSF3R* and *PTPN11* gain-of-function mutations are in line with other studies showing that mutations in cytokine and growth factor signaling pathways are frequently observed in ETP-ALL (Coustan-Smith et al. 2009; Neumann et al. 2013). *CSF3R* mutations are associated with response of chronic neutrophilic leukemia to JAK inhibitors such as ruxilitinib and provide another possible avenue for targeted therapy in ETP-ALL. One other recently reported ETP-ALL associated with the CSF3R I618T mutation (Maxson et al. 2013) also had a NOTCH1 NRR gain-of-function mutation (B Tyner, pers. comm.), indicating that this is a recurrent combination of mutations in this leukemia type. Also remarkable is the *DNMT3A* loss-of-function mutation, which was present in the ETP-ALL at homozygous dosage and persisted in the remission marrow at heterozygous dosage. The latter observation strongly suggests that this patient's ETP-ALL arose out of a background of clonal hematopoiesis, a common condition in older adults (Genovese et al. 2014; Jaiswal et al. 2014).

Unregulated Notch signaling drives normal hematopoietic stem cells to the CD4/CD8 double-positive stage of T-cell development (Pui et al. 1999); in contrast, Notch-mutated ETP-ALL cells, as in our case, arrest at what appears to correspond to an earlier stage of T-cell development during which cells maintain a potential for myeloid fate. We speculate that the inability of Notch to drive ETP-ALL cells to later stages of T-cell development stems from preexistence mutations (e.g., *DNMT3A* mutations) that skew the potential of hematopoietic stem cells toward myeloid fate. This may also explain the difference in response to Notch inhibition, as ETP-ALL cells represent a different cellular context than cortical-type T-ALL cells, and Notch effects are highly context dependent. Tests of this idea await development of genetically engineered mouse models with the combination of driver genes observed in this case.

## METHODS

### Chromatin Immunoprecipitation (ChIP)

ChIP was performed as described by Wang and colleagues (Wang et al. 2014) with the following modifications. Chromatin from formaldehyde-fixed cells (5 × 10^6^ cells per histone mark) was fragmented to a size range of 200–700 bases with a Branson 250 Sonifier. Solubilized chromatin was immunoprecipitated with an antibody against H3K27ac (2.5 μL; ActiveMotif, ab4729), and enrichment for H3K27ac was confirmed by dot blot as described (Ernst et al. 2011). Antibody–chromatin complexes were pulled down with protein G magnetic beads (Dynabead, 10003D), washed and then eluted. After cross-link reversal and proteinase K treatment, immunoprecipitated DNA was treated with RNase and purified with Agencourt AMPure XP (Beckman Coulter A63880). Libraries were prepared according to Illumina's instructions. ChIP DNA and input controls were sequenced with the Illumina HiSeq 2500 instrument. ChIP-seq data were processed and analyzed as described (Wang et al. 2014). Briefly, sequence reads were mapped to human genome build hg38 using Bowtie 2 (Langmead and Salzberg 2012). Uniquely mapped sequence reads were retained (Table 3). Genomic enrichment was identified using MACS 2.0 with broad peak calling under a false discovery threshold of 0.01 (Zhang et al. 2008). ChIP-seq traces were produced using the R package Gvis.

**Table 3.** Summary of sequencing studies

Sample	Sequencing type	Total reads	Aligned reads (%)	Average coverage
Aspirated marrow at presentation	Targeted-exon sequencing	1.6 × 10^7^	96.0	175-fold
Aspirated marrow at presentation	Whole-exon sequencing	1.3 × 10^9^	90.9	90-fold
Aspirated marrow at remission	Whole-exon sequencing	9.1 × 10^8^	91.7	124-fold
Blasts at presentation treated with DMSO	RNA-seq	1.3 × 10^9^	65.4	NA
Blasts at presentation treated with GSI	RNA-seq	7.9 × 10^9^	67.0	NA
Blasts at presentation	H3K27ac ChIP-seq	8.1 × 10^8^	98.0	NA

DMSO, dimethyl sulfoxide; RNA-seq, RNA sequencing; GSI, γ-secretase inhibitor; ChIP-seq, chromatin immunoprecipitation sequencing; NA, not applicable.

### Targeted Exome Sequencing (TES) and Whole-Exome Sequencing (WES)

TES was performed as described (Wagle et al. 2012)*.* WES was performed using DNA extracted from peripheral blood mononuclear cell pellets. Genomic DNA (250 ng) was subjected to shearing, barcoded sequencing library preparation, and hybrid capture as described (Fisher et al. 2011). Libraries were sequenced on the Broad Sequencing Platform (Cambridge, MA) with an Illumina HiSeq 2000 (San Diego, CA) using 76-bp paired-end reads. Sequencing data were aligned to the human reference genome hg19 using Picard tools (http://broadinstitute.github.io/picard/). Mutation calls were made on the WES samples with the mutation calling workflow in Firehose (https://www.broadinstitute.org/cancer/cga/Firehose) using Mutect (Cibulskis et al. 2013) with default parameters and enabled to run without matched normal DNA. Each mutation call made by Mutect was annotated using Oncotator (Ramos et al. 2015). Mutations that differed between the diagnostic and remission samples were curated from these lists. Likely driver mutations in *NOTCH1*, *PTPN11*, *DNMT3A*, and *CSF3R* were validated by aligning the original BAM files to hg19 and visualizing aligned reads in IGV (Robinson et al. 2011).

### Sanger Sequencing

Sanger sequencing of the region containing the *DNMT3A* c.1204C>T substitution was performed after PCR amplification of genomic DNA obtained from leukemic and remission marrow and from saliva following allogeneic hematopoietic stem cell transplantation using PFU Turbo DNA polymerase (Stratagene) with 100 μM of each primer in the presence of 10% DMSO under standard buffer and MgCl_2_ conditions. PCR conditions were 30 cycles of 95°C × 1 min, 55°C × 2 min, and 72°C × 3 min. PCR amplification products were sequenced directly by GeneWiz. PCR primers are given in Supplemental Table 1.

### RNA Sequencing (RNA-seq)

RNA-seq was performed on poly(A) selected RNA extracted from 1 × 10^6^ cultured leukemic cells (see below). Complementary DNA synthesis, barcoded library preparation and sequencing were performed as described (Wagle et al. 2014). RNA-seq data were analyzed using the PRADA pipeline as described (Bambury et al. 2015). Resultant data were transformed into gene-level expression values for downstream analysis (DeLuca et al. 2012).

### Cell Culture and Cell Culture Assays

Mononuclear cells were isolated from marrow using Ficoll gradients, washed, and resuspended in WIT medium in the presence of MS5 feeder cells expressing the Notch ligand DLL-1 for short-term culture assays, as described (Yost et al. 2013). To study effects of Notch inhibition, cultures were treated with the GSI DBZ (dibenzazepine, 1 μM) or DMSO vehicle (control). Notch reporter gene assays were performed in U2OS cells as described (Malecki et al. 2006). Antibody against β-actin was obtained from Sigma-Aldrich (A1978), and antibodies against activated NOTCH1 (D3B8) and MYC (9402) were from Cell Signaling Technologies.

### Mouse Xenograft Studies

Mononuclear cells from bone marrow aspirate were isolated on Ficoll gradients and injected by tail vein into NOD *scid* IL2R γ chain knockout (NSG) and NSG mice expressing IL-3, GM-CSF, and KIT ligand (NSG-S). Mice were followed for development of leukemia by monitoring the peripheral blood for appearance of blasts expressing human CD45. Immunohistochemical stains for activated NOTCH1 was performed in formalin-fixed paraffin-embedded sections as described (Kluk et al. 2013). All animal studies were performed using protocols approved by the Columbia University Medical Center IACUC.

## ADDITIONAL INFORMATION

### Ethics Statement

Human marrow, peripheral blood, and saliva samples were collected with informed consent and downstream analyses were performed under protocols 01-206 and 11-204 that were approved by the Institutional Review Board of the Dana-Farber/Harvard Cancer Center.

### Database Deposition and Access

Whole-exome sequencing, RNA sequencing, and ChIP-seq data are available through the NCBI Gene Expression Omnibus (GEO; http://www.ncbi.nlm.nih.gov/geo/) under accession number GSE71414. Point substitution variants were submitted to ClinVar (http://www.ncbi.nlm.nih.gov/clinvar/) (accession numbers SCV000239862–SCV000239865).

### Author Contributions

B.K., A.B., C.S.P., E.S., F.C.K., and M.M. contributed to analysis and interpretation of next-generation sequencing data. A.G. performed and interpreted TCRγ gene studies. D.M.D. interpreted flow cytometric findings. M.K. performed and interpreted immunohistochemical studies. L.P. performed and interpreted ex vivo assays using clinical samples and assays of the functional consequences of NOTCH1 mutations. S.C.B. provided structural models of the negative regulatory region of NOTCH1. A.L.K. oversaw xenograft studies. P.Z.-M. and D.J.D. contributed and reviewed the clinical features of the case. J.C.A. contributed micrographs of primary tumor specimens and xenografted tumors. All authors contributed to the writing of the manuscript.

### Funding

J.C.A. is supported by a grant from the National Institutes of Health (P01CA119070), a Leukemia & Lymphoma Society Specialized Center of Research (LLS SCOR) grant, and a grant from the William Lawrence and Blanche Hughes Foundation.

### Competing Interest Statement

The authors have declared no competing interest.

## Supplementary Material

Supplemental Material

## References

[KNOECHELMCS000539C1] Aster JC, Blacklow SC. 2012 Targeting the Notch pathway: twists and turns on the road to rational therapeutics. J Clin Oncol 30: 2418–2420. 22585704 10.1200/JCO.2012.42.0992

[KNOECHELMCS000539C2] Bambury RM, Bhatt AS, Riester M, Pedamallu CS, Duke F, Bellmunt J, Stack EC, Werner L, Park R, Iyer G, 2015 DNA copy number analysis of metastatic urothelial carcinoma with comparison to primary tumors. BMC Cancer 15: 242. 25886454 10.1186/s12885-015-1192-2 PMC4392457

[KNOECHELMCS000539C3] Cibulskis K, Lawrence MS, Carter SL, Sivachenko A, Jaffe D, Sougnez C, Gabriel S, Meyerson M, Lander ES, Getz G. 2013 Sensitive detection of somatic point mutations in impure and heterogeneous cancer samples. Nat Biotechnol 31: 213–219. 23396013 10.1038/nbt.2514 PMC3833702

[KNOECHELMCS000539C4] Coustan-Smith E, Mullighan CG, Onciu M, Behm FG, Raimondi SC, Pei D, Cheng C, Su X, Rubnitz JE, Basso G, 2009 Early T-cell precursor leukaemia: a subtype of very high-risk acute lymphoblastic leukaemia. Lancet Oncol 10: 147–156. 19147408 10.1016/S1470-2045(08)70314-0 PMC2840241

[KNOECHELMCS000539C5] Deftos ML, He YW, Ojala EW, Bevan MJ. 1998 Correlating notch signaling with thymocyte maturation. Immunity 9: 777–786. 9881968 10.1016/s1074-7613(00)80643-3 PMC2789684

[KNOECHELMCS000539C6] DeLuca DS, Levin JZ, Sivachenko A, Fennell T, Nazaire MD, Williams C, Reich M, Winckler W, Getz G. 2012 RNA-SeQC: RNA-seq metrics for quality control and process optimization. Bioinformatics 28: 1530–1532. 22539670 10.1093/bioinformatics/bts196 PMC3356847

[KNOECHELMCS000539C7] Ernst J, Kheradpour P, Mikkelsen TS, Shoresh N, Ward LD, Epstein CB, Zhang X, Wang L, Issner R, Coyne M, 2011 Mapping and analysis of chromatin state dynamics in nine human cell types. Nature 473: 43–49. 21441907 10.1038/nature09906 PMC3088773

[KNOECHELMCS000539C8] Fisher S, Barry A, Abreu J, Minie B, Nolan J, Delorey TM, Young G, Fennell TJ, Allen A, Ambrogio L, 2011 A scalable, fully automated process for construction of sequence-ready human exome targeted capture libraries. Genome Biol 12: R1. 21205303 10.1186/gb-2011-12-1-r1 PMC3091298

[KNOECHELMCS000539C9] Genovese G, Kahler AK, Handsaker RE, Lindberg J, Rose SA, Bakhoum SF, Chambert K, Mick E, Neale BM, Fromer M, 2014 Clonal hematopoiesis and blood-cancer risk inferred from blood DNA sequence. N Engl J Med 371: 2477–2487. 25426838 10.1056/NEJMoa1409405 PMC4290021

[KNOECHELMCS000539C10] Gordon WR, Roy M, Vardar-Ulu D, Garfinkel M, Mansour MR, Aster JC, Blacklow SC. 2009a Structure of the Notch1-negative regulatory region: implications for normal activation and pathogenic signaling in T-ALL. Blood 113: 4381–4390. 19075186 10.1182/blood-2008-08-174748 PMC2676092

[KNOECHELMCS000539C11] Gordon WR, Vardar-Ulu D, L'Heureux S, Ashworth T, Malecki MJ, Sanchez-Irizarry C, McArthur DG, Histen G, Mitchell JL, Aster JC, 2009b Effects of S1 cleavage on the structure, surface export, and signaling activity of human Notch1 and Notch2. PLoS One 4: e6613. 19701457 10.1371/journal.pone.0006613 PMC2726630

[KNOECHELMCS000539C12] Gutierrez A, Dahlberg SE, Neuberg DS, Zhang J, Grebliunaite R, Sanda T, Protopopov A, Tosello V, Kutok J, Larson RS, 2010 Absence of biallelic TCRγ deletion predicts early treatment failure in pediatric T-cell acute lymphoblastic leukemia. J Clin Oncol 28: 3816–3823. 20644084 10.1200/JCO.2010.28.3390 PMC2940399

[KNOECHELMCS000539C13] Herranz D, Ambesi-Impiombato A, Palomero T, Schnell SA, Belver L, Wendorff AA, Xu L, Castillo-Martin M, Llobet-Navas D, Cordon-Cardo C, 2014 A NOTCH1-driven MYC enhancer promotes T cell development, transformation and acute lymphoblastic leukemia. Nat Med 20: 1130–1137. 25194570 10.1038/nm.3665 PMC4192073

[KNOECHELMCS000539C14] Jaiswal S, Fontanillas P, Flannick J, Manning A, Grauman PV, Mar BG, Lindsley RC, Mermel CH, Burtt N, Chavez A, 2014 Age-related clonal hematopoiesis associated with adverse outcomes. N Engl J Med 371: 2488–2498. 25426837 10.1056/NEJMoa1408617 PMC4306669

[KNOECHELMCS000539C15] Kluk MJ, Ashworth T, Wang H, Knoechel B, Mason EF, Morgan EA, Dorfman D, Pinkus G, Weigert O, Hornick JL, 2013 Gauging NOTCH1 activation in cancer using immunohistochemistry. PLoS One 8: e67306. 23825651 10.1371/journal.pone.0067306 PMC3688991

[KNOECHELMCS000539C16] Kopan R, Ilagan MX. 2009 The canonical Notch signaling pathway: unfolding the activation mechanism. Cell 137: 216–233. 19379690 10.1016/j.cell.2009.03.045 PMC2827930

[KNOECHELMCS000539C38] Langmead B, Salzberg S. 2012 Fast gapped-read alignment with Bowtie 2. Nat Methods 9: 357–359. 22388286 10.1038/nmeth.1923 PMC3322381

[KNOECHELMCS000539C17] Larson RA, Dodge RK, Linker CA, Stone RM, Powell BL, Lee EJ, Schulman P, Davey FR, Frankel SR, Bloomfield CD, 1998 A randomized controlled trial of filgrastim during remission induction and consolidation chemotherapy for adults with acute lymphoblastic leukemia: CALGB study 9111. Blood 92: 1556–1564. 9716583

[KNOECHELMCS000539C18] Liu ZH, Dai XM, Du B. 2015 Hes1: a key role in stemness, metastasis and multidrug resistance. Cancer Biol Ther 16: 353–359. 25781910 10.1080/15384047.2015.1016662 PMC4622741

[KNOECHELMCS000539C19] Malecki MJ, Sanchez-Irizarry C, Mitchell JL, Histen G, Xu ML, Aster JC, Blacklow SC. 2006 Leukemia-associated mutations within the NOTCH1 heterodimerization domain fall into at least two distinct mechanistic classes. Mol Cell Biol 26: 4642–4651. 16738328 10.1128/MCB.01655-05 PMC1489116

[KNOECHELMCS000539C20] Maxson JE, Gotlib J, Pollyea DA, Fleischman AG, Agarwal A, Eide CA, Bottomly D, Wilmot B, McWeeney SK, Tognon CE, 2013 Oncogenic CSF3R mutations in chronic neutrophilic leukemia and atypical CML. N Engl J Med 368: 1781–1790. 23656643 10.1056/NEJMoa1214514 PMC3730275

[KNOECHELMCS000539C21] Mehra N, Lorente D, de Bono JS. 2015 What have we learned from exceptional tumour responses?: review and perspectives. Curr Opin Oncol 27: 267–275. 25811347 10.1097/CCO.0000000000000182

[KNOECHELMCS000539C22] Neumann M, Heesch S, Schlee C, Schwartz S, Gokbuget N, Hoelzer D, Konstandin NP, Ksienzyk B, Vosberg S, Graf A, 2013 Whole-exome sequencing in adult ETP-ALL reveals a high rate of DNMT3A mutations. Blood 121: 4749–4752. 23603912 10.1182/blood-2012-11-465138

[KNOECHELMCS000539C23] Pui JC, Allman D, Xu L, DeRocco S, Karnell FG, Bakkour S, Lee JY, Kadesch T, Hardy RR, Aster JC, 1999 Notch1 expression in early lymphopoiesis influences B versus T lineage determination. Immunity 11: 299–308. 10514008 10.1016/s1074-7613(00)80105-3

[KNOECHELMCS000539C24] Ramos AH, Lichtenstein L, Gupta M, Lawrence MS, Pugh TJ, Saksena G, Meyerson M, Getz G. 2015 Oncotator: cancer variant annotation tool. Hum Mutat 36: E2423–E2429. 25703262 10.1002/humu.22771 PMC7350419

[KNOECHELMCS000539C25] Real PJ, Tosello V, Palomero T, Castillo M, Hernando E, de Stanchina E, Sulis ML, Barnes K, Sawai C, Homminga I, 2009 γ-Secretase inhibitors reverse glucocorticoid resistance in T cell acute lymphoblastic leukemia. Nat Med 15: 50–58. 19098907 10.1038/nm.1900 PMC2692090

[KNOECHELMCS000539C26] Robinson JT, Thorvaldsdottir H, Winckler W, Guttman M, Lander ES, Getz G, Mesirov JP. 2011 Integrative genomics viewer. Nat Biotechnol 29: 24–26. 21221095 10.1038/nbt.1754 PMC3346182

[KNOECHELMCS000539C27] Shi J, Whyte WA, Zepeda-Mendoza CJ, Milazzo JP, Shen C, Roe JS, Minder JL, Mercan F, Wang E, Eckersley-Maslin MA, 2013 Role of SWI/SNF in acute leukemia maintenance and enhancer-mediated Myc regulation. Genes Dev 27: 2648–2662. 24285714 10.1101/gad.232710.113 PMC3877755

[KNOECHELMCS000539C28] Tartaglia M, Gelb BD. 2005 Noonan syndrome and related disorders: genetics and pathogenesis. Annu Rev Genomics Hum Genet 6: 45–68. 16124853 10.1146/annurev.genom.6.080604.162305

[KNOECHELMCS000539C29] Van Vlierberghe P, Ferrando A. 2012 The molecular basis of T cell acute lymphoblastic leukemia. J Clin Invest 122: 3398–3406. 23023710 10.1172/JCI61269 PMC3461904

[KNOECHELMCS000539C30] van Vlierberghe P, Meijerink JP, Lee C, Ferrando AA, Look AT, van Wering ER, Beverloo HB, Aster JC, Pieters R. 2006 A new recurrent 9q34 duplication in pediatric T-cell acute lymphoblastic leukemia. Leukemia 20: 1245–1253. 16673019 10.1038/sj.leu.2404247

[KNOECHELMCS000539C31] Wagle N, Berger MF, Davis MJ, Blumenstiel B, Defelice M, Pochanard P, Ducar M, Van Hummelen P, Macconaill LE, Hahn WC, 2012 High-throughput detection of actionable genomic alterations in clinical tumor samples by targeted, massively parallel sequencing. Cancer Discov 2: 82–93. 22585170 10.1158/2159-8290.CD-11-0184 PMC3353152

[KNOECHELMCS000539C32] Wagle N, Van Allen EM, Treacy DJ, Frederick DT, Cooper ZA, Taylor-Weiner A, Rosenberg M, Goetz EM, Sullivan RJ, Farlow DN, 2014 MAP kinase pathway alterations in BRAF-mutant melanoma patients with acquired resistance to combined RAF/MEK inhibition. Cancer Discov 4: 61–68. 24265154 10.1158/2159-8290.CD-13-0631 PMC3947296

[KNOECHELMCS000539C33] Wang H, Zang C, Taing L, Arnett KL, Wong YJ, Pear WS, Blacklow SC, Liu XS, Aster JC. 2014 NOTCH1-RBPJ complexes drive target gene expression through dynamic interactions with superenhancers. Proc Natl Acad Sci 111: 705–710. 24374627 10.1073/pnas.1315023111 PMC3896193

[KNOECHELMCS000539C34] Yashiro-Ohtani Y, Wang H, Zang C, Arnett KL, Bailis W, Ho Y, Knoechel B, Lanauze C, Louis L, Forsyth KS, 2014 Long-range enhancer activity determines Myc sensitivity to Notch inhibitors in T cell leukemia. Proc Natl Acad Sci 111: E4946–E4953. 25369933 10.1073/pnas.1407079111 PMC4246292

[KNOECHELMCS000539C35] Yost AJ, Shevchuk OO, Gooch R, Gusscott S, You MJ, Ince TA, Aster JC, Weng AP. 2013 Defined, serum-free conditions for in vitro culture of primary human T-ALL blasts. Leukemia 27: 1437–1440. 23246990 10.1038/leu.2012.337 PMC4704859

[KNOECHELMCS000539C36] Zhang Y, Liu T, Meyer CA, Eeckhoute J, Johnson DS, Bernstein BE, Nusbaum C, Myers RM, Brown M, Li W, 2008 Model-based analysis of ChIP-Seq (MACS). Genome Biol 9: R137. 18798982 10.1186/gb-2008-9-9-r137 PMC2592715

[KNOECHELMCS000539C37] Zhang J, Ding L, Holmfeldt L, Wu G, Heatley SL, Payne-Turner D, Easton J, Chen X, Wang J, Rusch M, 2012 The genetic basis of early T-cell precursor acute lymphoblastic leukaemia. Nature 481: 157–163. 22237106 10.1038/nature10725 PMC3267575

