# Supplementary material for: Complete hematologic response of early T-cell progenitor acute lymphoblastic leukemia to the γ-secretase inhibitor BMS-906024: genetic and epigenetic findings in an outlier case
Source: Cold Spring Harb Mol Case Stud. 2015 Oct;1(1):a000539. doi: 10.1101/mcs.a000539 (PMC4850884; doi:10.1101/mcs.a000539)
Supplement: Supplemental Material [file supp_1_1_a000539__index.html]

Supplemental Material 

# Complete hematologic response of early T-cell progenitor acute lymphoblastic leukemia to the γ-secretase inhibitor BMS-906024: genetic and epigenetic findings in an outlier case

## Supplemental Material

**Files in this Data Supplement:**

- Supplemental Figure1 Legend.docx
- Supplemental Table1.docx
- Supplemental Figure1.tif
