# Supplementary material for: Complete hematologic response of early T-cell progenitor acute lymphoblastic leukemia to the γ-secretase inhibitor BMS-906024: genetic and epigenetic findings in an outlier case
Source: Cold Spring Harb Mol Case Stud. 2015 Oct;1(1):a000539. doi: 10.1101/mcs.a000539 (PMC4850884; doi:10.1101/mcs.a000539)
Supplement: Supplemental Material [file supp_1.1.a000539_Supplemental_Table1.docx]

**Supplemental Table 1. PCR primers used to amplified gene segments containing driver mutations**

| **Gene** | **Forward primer** | **Reverse primer** |
| --- | --- | --- |
| *NOTCH1* | 5'-GACCAGTACTGCAAGGACCACTTC | 5'-TCCTCGCGGCCGTAGTAGGGGAAG |
| *PTPN11* | 5'-GGCATTTTGAGACATCAGGCAGTG | 5'-AGGCTAGAAATGTATGGTCAGAAAACGC |
| *DNMT3A* | 5'-CCACCCTCACTACTCAGAGTCTGGCC | 5'-CCTTGGCAGCCCTCCCTAAGCATGGC |
| *CSF3F* | 5'-CCGTACACCCCTCCCAGCCGCCATCC | 5'-TGATGTGTAGCTCCGACCCCTCTGC |
