# Supplementary figures and images for: Complete hematologic response of early T-cell progenitor acute lymphoblastic leukemia to the γ-secretase inhibitor BMS-906024: genetic and epigenetic findings in an outlier case
Source: Cold Spring Harb Mol Case Stud. 2015 Oct;1(1):a000539. doi: 10.1101/mcs.a000539 (PMC4850884; doi:10.1101/mcs.a000539)

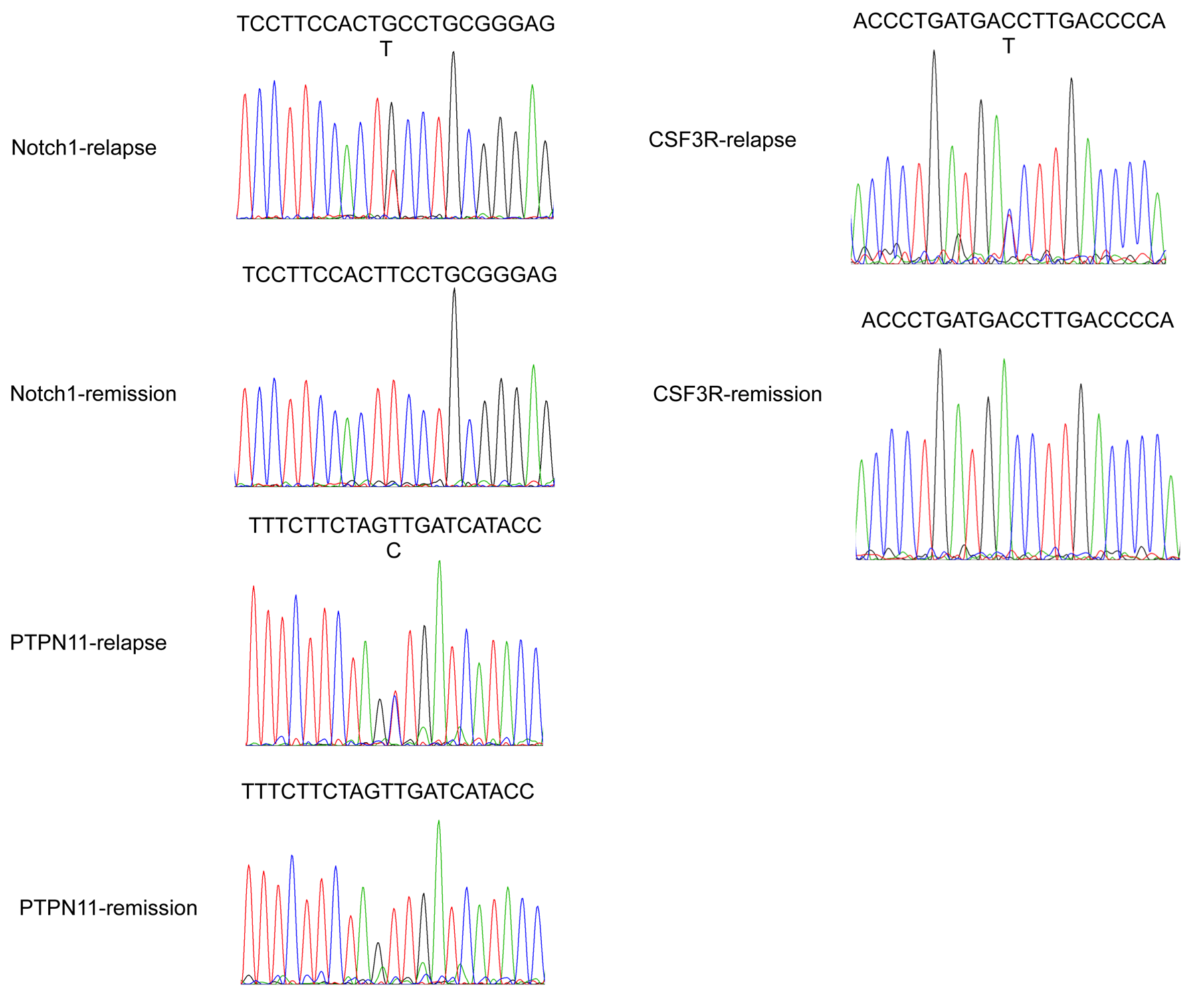

Supplement: Supplemental Material [file supp_1.1.a000539_Supplemental_Figure1.tif]
